# Gelam honey attenuated radiation-induced cell death in human diploid fibroblasts by promoting cell cycle progression and inhibiting apoptosis

**DOI:** 10.1186/1472-6882-14-108

**Published:** 2014-03-24

**Authors:** Tengku Ahbrizal Farizal Tengku Ahmad, Faizul Jaafar, Zakiah Jubri, Khairuddin Abdul Rahim, Nor Fadilah Rajab, Suzana Makpol

**Affiliations:** 1 Department of Biochemistry, Faculty of Medicine, Universiti Kebangsaan Malaysia, Jalan Raja Muda Abdul Aziz, Kuala Lumpur 50300, Malaysia; 2 Division of Agrotechnology and Biosciences, Malaysian Nuclear Agency, Bangi, Kajang 43000, Malaysia; 3 Faculty of Health Sciences, Universiti Kebangsaan Malaysia, Jalan Raja Muda Abdul Aziz, Kuala Lumpur 50300, Malaysia

**Keywords:** Gelam honey, Gamma-irradiation, Cell cycle progression, Apoptosis

## Abstract

**Background:**

The interaction between ionizing radiation and substances in cells will induce the production of free radicals. These free radicals inflict damage to important biomolecules such as chromosomes, proteins and lipids which consequently trigger the expression of genes which are involved in protecting the cells or repair the oxidative damages. Honey has been known for its antioxidant properties and was used in medical and cosmetic products. Currently, research on honey is ongoing and diversifying. The aim of this study was to elucidate the role of Gelam honey as a radioprotector in human diploid fibroblast (HDFs) which were exposed to gamma-rays by determining the expression of genes and proteins involved in cell cycle regulation and cell death.

**Methods:**

Six groups of HDFs were studied viz. untreated control, irradiated HDFs, Gelam honey-treated HDFs and HDF treated with Gelam honey pre-, during- and post-irradiation. HDFs were treated with 6 mg/ml of sterilized Gelam honey (w/v) for 24 h and exposed to 1 Gray (Gy) of gamma-rays at the dose rate of 0.25 Gy/min.

**Results:**

Our findings showed that, gamma-irradiation at 1 Gy up-regulated *ATM, p53, p16*^*ink4a*^ and *cyclin D1* genes and subsequently initiated cell cycle arrest at G_0_/G_1_ phase and induced apoptosis (p < 0.05). Pre-treatment with Gelam honey however caused down regulation of these genes in irradiated HDFs while no significant changes was observed on the expression of *GADD45* and *PAK* genes. The expression of ATM and p16 proteins was increased in irradiated HDFs but the *p53* gene was translated into p73 protein which was also increased in irradiated HDFs. Gelam honey treatment however significantly decreased the expression of ATM, p73, and p16 proteins (p < 0.05) while the expression of cyclin D1 remained unchanged. Analysis on cell cycle profile showed that cells progressed to S phase with less percentage of cells in G_0_/G_1_ phase with Gelam honey treatment while apoptosis was inhibited.

**Conclusion:**

Gelam honey acts a radioprotector against gamma-irradiation by attenuating radiation-induced cell death.

## Background

Ionizing radiation is known to cause DNA damage which leads to cell cycle arrest at G_0_/G_1_ phase. The G_1_ phase is critical for cells to get ready for DNA synthesis at S phase [1], while at G_2_ phase cells make final preparation before division during mitosis [2]. Defects in both phases may allow cells with damaged DNA to enter mitosis phase. Two discrete cell cycle checkpoints are present at G1/S or G2/M phases. Upon radiation exposure, these checkpoints will be activated which provides time for DNA repair and thereby promote genomic stability [3]. However in certain condition, the DNA damage is irreparable and cells will either enter a permanent growth arrest at G_0_ phase [4] or undergo apoptosis [5].

Cell cycle progression depends on the integration of growth control pathways with cell cycle mechanism. Ionizing radiation can trigger formation of reactive oxygen species (ROS), which directly attacked many biomolecules in the cell including enzymes [6], lipids and proteins as well as caused nucleotide damage, single-strand breaks (SSBs) or double-strand breaks (DSBs) in DNA molecules [7]. The most lethal DNA lesion caused by ionizing radiation is double-strand breaks. Damaged DNA is detected by *ataxia telangiectasia mutated (ATM)* gene. Activation of *ATM* gene leads to cell cycle arrest or induction of apoptosis through downstream *p53* gene [8,9]. The *p53* gene is known as the guardian of genomic stability or tumor suppressor gene [10]. Besides p53, another group member of tumor suppressor protein is p73. *p53* and *p73* genes are located at different loci in the chromosome [11,12]. Both proteins however shared the same characteristic especially at the transactivation domain (30%), DNA binding domain (60%) and oligomerization domain (37%) [13,14]. p73 protein also shared the same signaling pathway as p53 which initiated cell cycle arrest and induced apoptosis when DNA damage occurs in the cells [15].

In normal condition, cyclin D binds to cyclin-dependent kinases (CDKs) 4/6 and allows cells to progress to S phase by phosphorylating the retinoblastoma protein (pRb). p16, a cyclin dependent kinase inhibitor (CDKI) is involved in regulating cell cycle progression. Activated p16^ink4a^ binds to and inhibits CDK 4/6, which separated cyclin D1-CDK4/6 complex. As a result pRB is dephosphorylated which leads to cell cycle arrest [16]. Previous study showed that both p53 and p16^ink4a^ have parallel functions which inhibit cell cycle progression upon binding to CDK4/6. It also has been reported that decreased expression of p53 protein can lead to up-regulation of p16^ink4a^[17].

Cell cycle also can be arrested at G2/M phase when p53 activates GADD45 which inhibits formation of cyclin B1-cdc2 complex [18,19]. GADD45 has three isoforms; GADD45α, GADD45β and GADD45γ [20,21]. Each isoform will be activated by different oxidant inducer and cell types [22].

Although cell defense system can detect and repair damaged DNA, there is a limitation in DNA repairing process particularly when the damaged DNA is extensive. Several studies have been conducted to determine a potential radioprotectant agent [23-25] which can prevent radiation-induced DNA damage.

Honey is derived from floral nectar and produced by honeybee which contains mixture of sugars such as fructose and glucose [26] and other constituents such as phenolic compound, mineral, protein, free amino acid, enzyme and vitamin [27,28]. The presence of various constituents in honey give rise to its different biological properties such as anti-cancer, anti-microbial, promote wound healing, anti-inflammatory, anti-diabetic and antioxidant [29-33]. Gelam honey is one of Malaysian monofloral honeys produced by *Apis mellifera* from *Melaleuca cajuputi* nectar and pollen [34]. Gelam honey has high total phenolic content and high concentration of flavonoid [34] and our previous study showed it possessed antioxidant property [26,35]. Total phenolic content represents by the presence of polyphenols, is correlated with its antioxidant activity. Flavonoid is another phenolic compound presents in honey that has been studied extensively and shows best antioxidant effect [36] as it scavenges free radicals and prevents DNA damage [37].

There are several known phenolic compounds that have been found in Gelam honey such as ascorbic acid, catecilin, benzoic acid, naringenin, luteolin, kaempferol and apigenin [34]. Our previous study showed that Gelam honey acts as a radioprotectant agent by protecting the DNA and enhancing cell survival rate in gamma-irradiated human diploid fibroblasts (HDFs) [38]. Besides, Gelam honey also maintained catalase (CAT) and superoxide dismutase (SOD) enzyme activities in HDFs when exposed to 1 Gy of gamma-rays [39]. Although the ability of Gelam honey as radioprotectant agent has been shown, the signaling pathways involved remain unclear.

In the present study we elucidated the molecular mechanism of Gelam honey in preventing radiation-induced cell death by determining the expression of ATM, p53/73, p16^ink4a^, cyclin D1, GADD45 and PAK2 genes and proteins in human diploid fibroblasts (HDFs). Cell cycle profile and apoptosis were also evaluated to fascilitate better understanding of the molecular events following gamma-irradiation and to elucidate the protective effects of Gelam honey in human diploid fibroblasts. Skin fibroblast cells were used in this study because skin is the largest external organ highly exposed to ionizing radiation while fibroblast cells are the most common cells in connective tissue that are responsible for producing tissue elements and sensitive to ionizing radiation [39].

## Methods

### Sterilization of gelam honey

Malaysian monofloral Gelam honey is produced by *Apis mellifera*, and the major nectar and pollen collected by the bees is from the plant *Melaleuca cajuputi* Powell, which is known locally as the “Gelam tree”. It was purchased from the Department of Agriculture, Batu Pahat, Johor, Malaysia. Gelam honey was packed in tight cap plastic bottles and placed in a box before sending to SINAGAMA, Malaysian Nuclear Agency. The sterilization process was carried out using Cobalt-60 source (Model JS10000, Atomic Energy of Canada Ltd, Ontario, Canada). The box which contained Gelam honey was carried into a gamma-radiation chamber and circled the Cobalt-60 source for 5 times to reach the dose of 25 kiloGray (kGy). The dose was automatically calculated by Cobalt-60 machine. The irradiated Gelam honey was then kept in the dark at room temperature.

### Cell culture protocol

Primary HDFs were derived from foreskins of three 9 to 12 year-old boys after circumcision. Written informed consents were obtained from parents of all subjects. The samples were aseptically collected and washed several times with 75% alcohol and phosphate buffered saline (PBS) containing 1% antibiotic-antimycotic solution (PAA, Pasching, Austria). After removing the epidermis, the pure dermis was cut into small pieces and transferred into a falcon tube containing 0.03% collagenase type I solution (Worthington Biochemical Corporation, Lakewood, NJ, USA). The pure dermis was digested in an incubator shaker at 37°C for 6–12 h. Cells were then rinsed with PBS before being cultured in Dulbecco Modified Eagle Medium (DMEM, Flowlab™, North Ryde, Australia), 10% fetal bovine serum (PAA, Austria), 10,000 μg/mL penicillin/streptomycin (Gibco, Grand Island, NY, USA), 250 μg/mL amphotericin B (PAA, Austria), 100 mg/mL gentamycin (PAA, Austria) and incubated in 5% CO_2_ atmosphere at 37°C. This research has been approved by the National University of Malaysia Ethical Committee (Approval Project Code: FF-287-2009).

### Gelam honey treatment protocol

HDFs were treated with 6 mg/mL of sterilized Gelam honey (w/v) with 24 h incubation. The concentration of Gelam honey was selected based on previous cytotoxicity study [38]. There were six different groups of HDFs viz. non-irradiated and non-honey treated HDFs (untreated control), irradiated HDFs and HDFs treated with Gelam honey alone. The other three groups were HDFs treated with Gelam honey pre-, during- and post-irradiation.

### Exposure to gamma-irradiation

HDFs were exposed to gamma-rays at 1 Gy using the ELDORADO 8 cobalt-60 source (Atomic Energy of Canada Ltd, Ontario, Canada) at the Secondary Standard Dosimetry Laboratory (SSDL, Malaysian Nuclear Agency). For 1 Gy of gamma-rays exposure, the dosage rate was calculated to be 0.25 Gy/min on the day the cells were irradiated. The cell culture flask was placed in the radiation chamber and the distance between the radiation source and the cell culture flask was 80 cm.

### Primer design

Primers for human *ATM*: 5′-ccg tga tga cct gag aca ag-3′ (forward) and 5′-aac acc act tcg ctg aga gag-3′ (reverse)*; p53*: 5′-gga aga gaa tct ccg caa gaa-3′ (forward) and 5′-agc tct cgg aac atc tcg aag-3′ (reverse); *p16*^*ink4a*^: 5′-agt gag ggt ttt cgt ggt tca c-3′ (forward) and 5′-cca tca tca tga cct ggt ctt cta-3′ (reverse); *cyclin D1*:5′-aga cct tcg ttg ccc tct gt-3′ (forward) and 5′-cag tcc ggg tca cac ttg at-3′ (reverse); *GADD45*: 5′-cca aga tgc cac aga tga ttg-3′(forward) and 5′-act cct tgg gtc cac ctg gta-3′(reverse); *PAK2*: 5′-gat ggc acc aga ggt ggt ta-3′(forward) and 5′-tcc cga aat att ggg gaa ag-3′(reverse) and housekeeping gene, *GADPH*: 5′-tcc ctg agc tga acg gga ag-3′ (forward) and 5′-gga gga gtg ggt gtc gct gt-3′ (reverse) were designed from listed NIH GenBank database using Primer 3 software and blasted against GenBank database sequences.

### Total RNA extraction

Total RNA was extracted from HDFs using TRI Reagent (Molecular Research Center, Cincinnati, OH, USA). Polyacryl Carrier (Molecular Research Center, Cincinnati, OH, USA) was added to precipitate the RNA before centrifuging to collect the RNA pellet. The RNA pellet was then washed with 75% ethanol and allowed to dry before adding RNase and DNase free distilled water to dissolve the pellet. All total RNA extracts were kept at −80°C prior to use.

### Real-time quantitative RT-PCR analysis

Quantitative RT-PCR reaction was carried out using iScript One-Step RT-PCR Kit with SYBR Green (Bio-Rad, Hercules, CA, USA). Master mix of RNA extract, nuclease-free H_2_O, 2X SYBR Green and reverse transcript (RT) solution was aliquoted into each reaction tube which contained forward and reverse primers. Reactions were conducted using iQ5 Bio-Rad iCycler with the following reaction profile; cDNA synthesis for 20 min at 50°C, reverse transcriptase inactivation for 4 min at 95°C and 38 cycles of PCR amplification of 10 sec at 95°C and 30 sec at 61°C. Melt curves were analysed at 95°C for 1 min. The expression level of each targeted gene was normalized to glyceraldehyde 3-phosphate dehydrogenase (GAPDH) gene as an internal reference [40]. Agarose gel electrophoresis was performed for confirmation of the PCR products. Relative expression value of target genes was calculated based on the 2^−ΔΔCt^ method of relative quantification [41] by the following equation:

$$\mathrm{Relative}\;\mathrm{expression}\;\mathrm{value}=2^{\mathrm{Ct}\;\mathrm{value}\;\mathrm{of}\;\mathrm{GAPDH}-\mathrm{Ct}\;\mathrm{value}\;\mathrm{gene}\;\mathrm{of}\;\mathrm{interest}}$$

### Protein extraction

HDFs were harvested and centrifuged to collect the cell pellet. The pellet was resuspended in cold PBS (50 mM, pH 7.0) and incubated in ice for 10 min before centrifuged. Lysis buffer [complete mini EDTA-free (Roche, Indianapolis, USA) in RIPA buffer (Sigma, St. Louis, MO, USA)] was added followed by incubation at 4°C for 30 min. Suspension was then centrifuged to collect the supernatant which contains the enzyme extract. Protein concentration was determined by Bradford assay using bovine serum albumin as a standard protein. The total protein was expressed in mg/mL.

### Western blot analysis

The protocol was carried out as described by WesternBreeze® Chemiluminescent Western Blot Immunodetection Kit (USA). Master mix was prepared by mixing the protein extract with 4X NuPAGE LDS sample buffer (Invitrogen, USA) and NuPAGE reducing agent (Invitrogen, USA). Ultrapure water was added up to 10 μl of final volume. The master mix was then heated at 70°C for 10 min prior to use. The p53 protein was determined using Bis-Tris (4-12%) gel (Invitrogen, USA) and Magic Mark (Invitrogen, USA) as a marker. Electrophoresis was carried out at 200 V for 35 min using Invitrogen Electrophoresis Set (USA). Meanwhile, ATM protein was determined using Tris-acetate (3-8%) gel (Invitrogen, USA) and High Mark (Invitrogen, USA) as a marker. Electrophoresis was carried out at 150 V for 1 h using Invitrogen Electrophoresis Set (USA). Protein bands from the gel were transferred to a nitrocellulose membrane (Invitrogen, USA). The membrane was incubated with p53 antibody for 1 h and overnight for ATM antibody. Primary mouse monoclonal antibodies used in this study were ATM (Ab 78: Abcam, USA), p53 (SC-98: Santa Cruz, USA), p16 (SC-9968: Santa Cruz, USA), cyclin D1 (SC-20044; Santa Cruz, USA) and internal control β-actin (SC-69879: Santa Cruz, USA). The membrane was later incubated with secondary mouse antibodies for 1 h and the antibody reaction was then revealed by chemiluminescence detection. The bands present on the membrane were visualized using Gel Documentation System (Alpha Innotech, USA) and protein density was determined by Total lab Software Version 1.11 (Alpha Innotech, USA). The amount of protein of interest was expressed in arbitrary unit by normalizing the protein density with protein density of β-actin.

### Cell cycle analysis

HDFs were harvested at desired time points after trypsinization and were rinsed 3 times with buffer solution with adjusted concentration 1×10^6^ cells/ml and prepared using CycleTEST™ PLUS DNA Reagent Kit (Becton Dickinson, USA) according to the manufacturer’s instruction. Cell cycle progression was determined using CycleTEST™ PLUS DNA Reagent Kit (Becton Dickinson, USA). Approximately 60% confluenced HDFs were collected and rinsed 3 times with buffer solution with adjusted concentration 1×10^6^ cells/ml and prepared according to the manufacturer’s instruction. Cell cycle status was analyzed by flow cytometer using propidium iodide (PI) as a specific fluorescent dye probe. The PI fluorescence intensity of 10,000 cells was measured for each sample using a Becton–Dickinson FACS Calibur Flow Cytometer. The fraction of cells in each phase of the cell cylce was quantified using a ModFit Software.

### Apoptosis determination

Measurement of Annexin V-FITC was carried out using Annexin V-FITC Apoptosis Detection Kit II (Becton Dickinson, Pharmigen™, San Diego, CA, USA) according to the manufacturer’s instructions. Approximately 60% confluenced HDFs were collected and washed twice with cold PBS and resuspended in 1X binding buffer. Then 5 μl of Annexin V-FITC and 5 μl of PI staining solution were added, followed by incubation for 15 min in the dark at room temperature (25°C). Finally, cells were suspended in 1× binding buffer and analyzed within 1 h by FACS Calibur Flow Cytometer (Becton Dickinson, San Jose, CA, USA).

### Statistical analysis

All experiments were carried out in duplicate with 3 independent cultures. Data are reported as means ± SD and comparison between groups was made by ANOVA. *p* < 0.05 was considered statistically significant.

## Results

### Cell cycle progression

**Figure 1:**
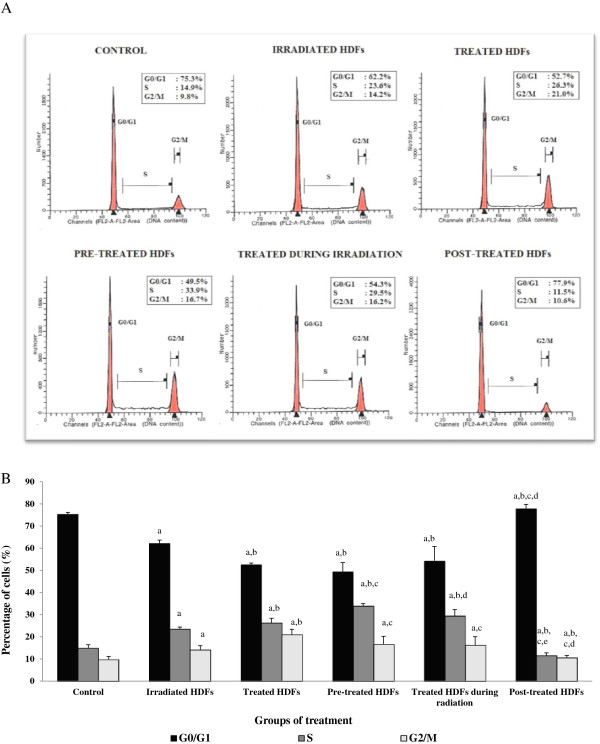
**Flow cytometry analysis of cell cycle progression in untreated control, irradiated and Gelam honey-treated HDFs (A).** Quantitative analysis of cell cycle progression in untreated control, irradiated and Gelam honey-treated HDFs. Cell population in the G_0_/G_1_ phase was significantly decreased while S phase cells increased in irradiated and Gelam honey-treated HDFs **(B)**. ^a^Denotes p < 0.05 compared to untreated control, ^b^p < 0.05 compared to irradiated HDFs, ^c^p < 0.05 compared to Gelam honey-treated HDFs, ^d^p < 0.05 compared to HDFs treated during irradiation. Comparison was done between HDFs in the same phase of cell cycle. Data are expressed as mean ± SD (n = 6).

Analysis on cell cycle profile showed that HDFs population in G_o_/G_1_ phase was significantly decreased while S phase and G_2_/M phase cells increased in irradiated and Gelam honey-treated HDFs as compared to untreated control HDFs (Figure 1A, 1B) (p < 0.05). Irradiated HDFs pre-treated with Gelam honey or treated with Gelam honey during irradiation showed decreased percentage of cells in G_0_/G_1_ phase with significant increased in S phase cells as compared to irradiated HDFs (p < 0.05) without Gelam honey treatment. Cells treated with Gelam honey after irradiation with gamma-rays however showed higher percentage of cells in G_0_/G_1_ phase with less cells in S and G_2_/M phases as compared to untreated control and irradiated HDFs (p < 0.05).

### Apoptotic changes detected by Annexin V-FITC

**Figure 2:**
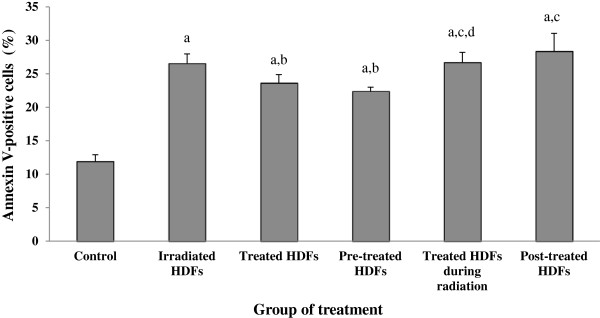
**Percentage of cells at early apoptotic stage demonstrated by FITC**^**+**^**/PI**^**−**^**.** The percentage of cells at early apoptotic stage was significantly increased in irradiated and Gelam honey-treated HDFs as compared to untreated control. Irradiated HDFs pretreated with Gelam honey showed decreased percentage of cells at early apoptotic stage as compared to irradiated HDFs. ^a^Denotes p < 0.05 compared to untreated control, ^b^p < 0.05 compared to irradiated HDFs, ^c^p < 0.05 compared to Gelam honey-treated HDFs, ^d^p < 0.05 compared to pre-treated HDFs, Data are expressed as mean ± SD (n = 6).

The percentage of cells at early apoptotic stage was significantly increased in irradiated and Gelam honey-treated HDFs as compared to control (Figure 2) (p < 0.05). Irradiated HDFs pre-treated with Gelam honey showed decreased percentage of cells at early apoptotic stage as compared to irradiated HDFs (p < 0.05).

### Analysis of ATM, p53, p16^ink4a^, cyclin D1, GADD45 and PAK2 genes expression

**Figure 3:**
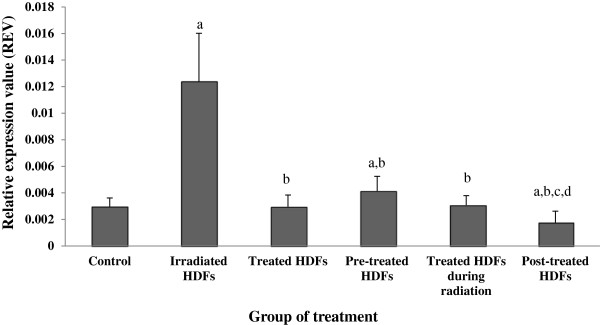
**Relative expression value of *ATM *gene in different treatment groups of HDFs. **^a^Denotes p < 0.05 compared to untreated control, ^b^p < 0.05 compared to irradiated HDFs, ^c^p < 0.05 compared to Gelam honey-treated HDFs, ^d^p < 0.05 compared to HDFs treated during irradiation. Data are expressed as mean ± SD (n = 6).

Gene expression analysis showed that the *ATM* gene was significantly up-regulated in irradiated HDFs as compared to untreated control (Figure 3) (p < 0.05). In contrast, *ATM* gene was significantly down-regulated in irradiated HDFs treated with Gelam honey before, during or after irradiation with gamma-rays (p < 0.05) as compared to irradiated HDFs without Gelam honey treatment.

**Figure 4:**
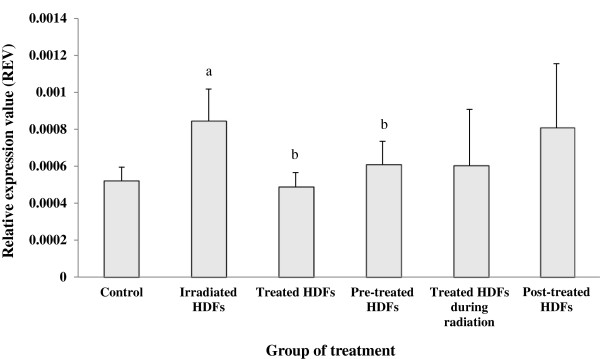
**Relative expression value of *p53 *gene in different treatment groups of HDFs. **^a^Denotes p < 0.05 compared to untreated control, ^b^p < 0.05 compared to irradiated HDFs. Data are expressed as mean ± SD (n = 6).

Irradiation with gamma-rays caused a significant increased in the expression of *p53* gene in HDFs (p < 0.05) (Figure 4) as compared to untreated control while treatment with Gelam honey down regulated the *p53* gene. Similar down-regulation of *p53* gene was observed in irradiated HDFs pre-treated with Gelam honey (p < 0.05) as compared to irradiated HDFs.

**Figure 5:**
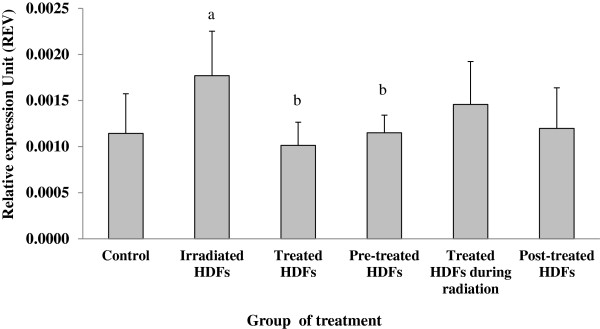
**Relative expression value of *p16***^***ink4a ***^**gene in different treatment groups of HDFs. **^a^Denotes p < 0.05 compared to untreated control, ^b^p < 0.05 compared to irradiated HDFs. Data are expressed as mean ± SD (n = 6).

The *p16*^*ink4a*^ gene was significantly up-regulated in irradiated HDFs as compared to untreated control (p < 0.05) (Figure 5). Down-regulation of *p16*^*ink4a*^ gene was observed in HDFs treated with Gelam honey alone and irradiated HDFs pre-treated with Gelam honey as compared to irradiated HDFs (p < 0.05).

**Figure 6:**
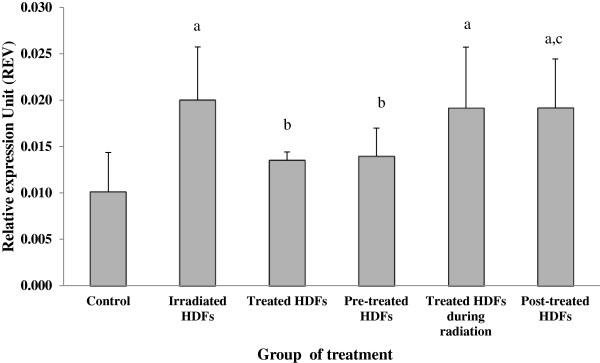
**Relative expression value of *cyclin D1 *gene in different treatment groups of HDFs. **^a^Denotes p < 0.05 compared to untreated control, ^b^p < 0.05 compared to irradiated HDFs, ^c^p < 0.05 compared to Gelam honey-treated HDFs. Data are expressed as mean ± SD (n = 6).

Similarly, *cyclin D1* gene was up-regulated in irradiated HDFs as compared to untreated control (p < 0.05) (Figure 6). Both HDFs treated with Gelam honey and irradiated HDFs pre-treated with Gelam honey showed down-regulation of *cyclin D1* gene as compared to irradiated HDFs. HDFs treated with Gelam honey during radiation and post-irradiation however showed significant up-regulation of *cyclin D1* gene as compared to untreated control (p < 0.05).

**Figure 7:**
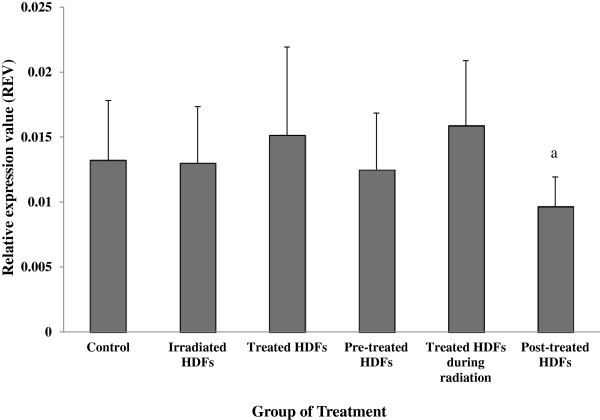
**Relative expression value of *GADD45 *gene in different treatment groups of HDFs. **^a^Denotes p < 0.05 compared to untreated control. Data are expressed as mean ± SD (n = 6).

**Figure 8:**
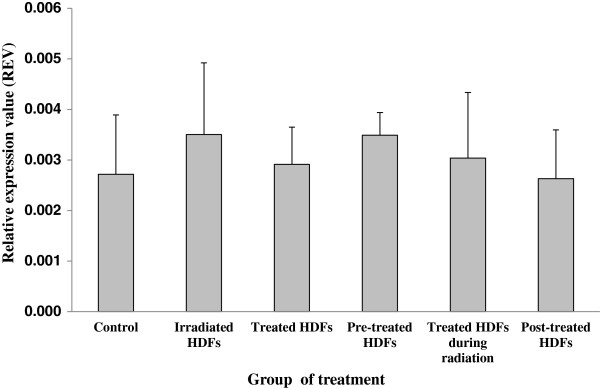
**Relative expression value of *PAK2 *gene in different treatment groups of HDFs.** Data are expressed as mean ± SD (n = 6).

HDFs treated with Gelam honey after irradiation with gamma-rays showed significant down-regulation of *GADD45* gene as compared to untreated control (p < 0.05) (Figure 7). No significant changes however was observed in other treatment groups. Similarly, no significant changes was observed on the expression of *PAK2* gene in all treatment groups (Figure 8).

### Analysis of ATM, p73, p16 and cyclin D1 protein expression

**Figure 9:**
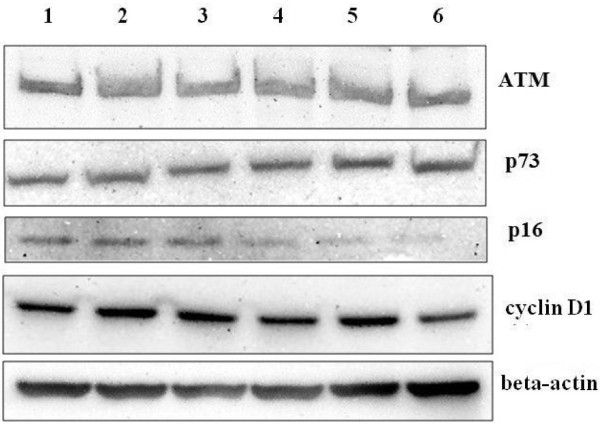
Representative Western blot of ATM, p73, p16, cyclin D1 and β-actin proteins in HDFs (lane 1: untreated control; lane 2: irradiated HDFs; lane 3: Gelam honey-treated HDFs; 4: pre-treated HDFs; 5: treated HDFs during irradiation; 6: post-treated HDFs.

**Figure 10:**
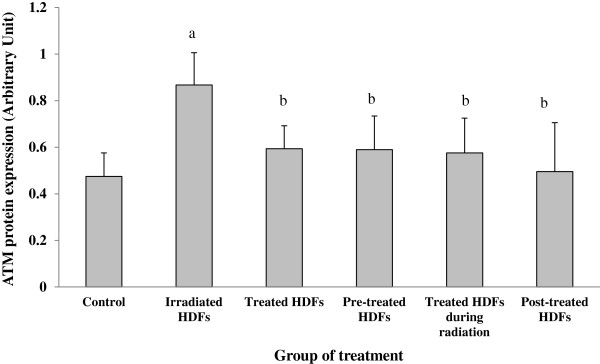
**Expression of ATM protein in different treatment groups of HDFs. **^a^Denotes p < 0.05 compared to control, ^b^p < 0.05 compared to irradiated HDFs. Data are expressed as mean ± SD (n = 6).

Figure 9 shows the representative protein bands determined by Western blot. Gamma-irradiation significantly increased the expression of ATM protein in HDFs as compared to untreated control (p < 0.05) (Figure 10). In contrast, Gelam honey treatment pre-, during- and post-irradiation decreased the expression ATM protein in irradiated HDFs as compared to irradiated HDFs (p < 0.05) without Gelam honey treatment.

**Figure 11:**
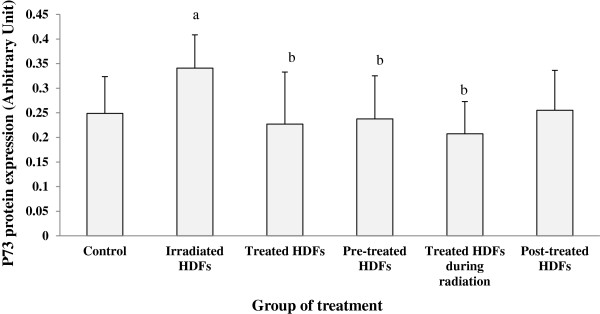
**Expression of p73 protein in different treatment groups of HDFs. **^a^Denotes p < 0.05 compared to control, ^b^p < 0.05 compared to irradiated HDFs. Data are expressed as mean ± SD (n = 6).

Similar increased on the expression of p73 protein was observed in irradiated HDFs as compared to untreated control (p < 0.05) (Figure 11). Gelam honey treatment pre- and during-irradiation decreased the expression of p73 protein as compared to irradiated HDFs (p < 0.05).

**Figure 12:**
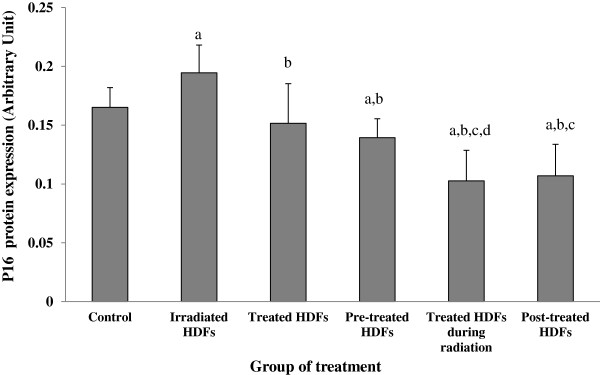
**Expression of p16 protein in different treatment groups of HDFs. **^a^Denotes p < 0.05 compared to control, ^b^p < 0.05 compared to irradiated HDFs, ^c^p < 0.05 compared to Gelam honey-treated HDFs, ^d^p < 0.05 compared to pre-treated HDFs. Data are expressed as mean ± SD (n = 6).

The expression of p16 protein was increased in irradiated HDFs as compared to untreated control (p < 0.05) (Figure 12). Gelam honey treatment pre-, during- and post-irradiation decreased the expression p16 protein in irradiated HDFs as compared to irradiated HDFs (p < 0.05).

**Figure 13:**
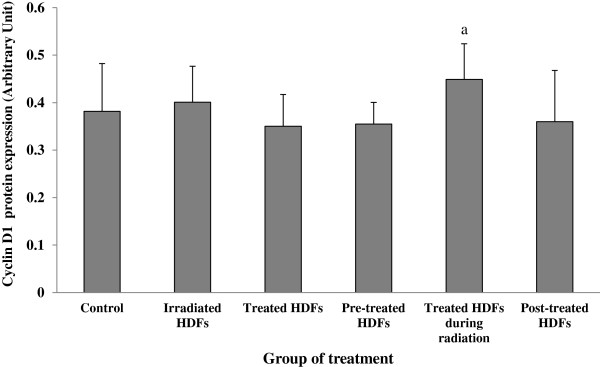
**Expression of Cyclin D1 protein in different treatment groups of HDFs. **^a^Denotes p < 0.05 compared to control. Data are expressed as mean ± SD (n = 6).

HDFs treated with Gelam honey during irradiation with gamma-rays showed significant increased in cyclin D1 protein expression as compared to untreated control (p < 0.05) (Figure 13). No significant changes however was observed in other treatment groups.

## Discussion

Several pathways are involved in maintaining genetic integrity when cells are exposed to ionizing radiation. A common cellular response to radiation injuries is the activation of cell cycle checkpoints to stop cell cycle progression. The presence of damaged DNA may initiate growth arrest at G_0_/G_1_ and G_2_/M phases or apoptosis [42]. In this study, the effect of gamma-irradiation on HDFs was elucidated by determining the expression of genes and proteins involved in the regulation of cell cycle. The presence of early apoptotic cells as a result of cellular damages was also evaluated.

Previous studies showed that ionizing radiation initiated the activation of *ATM* gene by auto phosphorylation after induction of DNA double strand breaks (DSBs) [43]. A study done by Zhang et al. [44] indicated that ionizing radiation up-regulated ATM in human fibroblast cells when exposed to gamma-rays, whereas Warter et al. showed that gamma-irradiation activated p53 in fibroblast and keratinocyte cells [45]. Thus gamma-irradiation caused damage to the DNA which consequently results to up-regulation of ATM and p53 in HDFs. Activation of ATM and p53 in irradiated HDFs leads to cell cycle arrest at G_0_/G_1_ phase and induced apoptosis. Similar findings have been shown by Antoccia et al. which reported that fibroblast cells exposed to 1–4 Gy of protons- and X/gamma-radiation failed to commit to S phase of the cell cycle and remained arrested in G_1_ phase for several days [46].

Our results showed that eventhough the percentage of cells in G_0_/G_1_ phase was significantly higher in untreated control cells but the ATM and p73 gene and protein were not up-regulated. Our previous findings showed that DNA damage was lower and cell survival rate was significantly increased in untreated control HDFs as compared to irradiated HDFs [38]. Therefore, the higher percentage of cells in G_0_/G_1_ phase in the untreated control HDFs observed in this study could be due to the presence of over confluenced cells which did not undergo cell proliferation and as a result cells remained in G_0_/G_1_ phase.

We also found that gamma-rays irradiation induced programmed cell death or apoptosis in HDFs. The induction of apoptosis could be explained by activation/up-regulation of p53 in the presence of irreparable radiation-induced damaged DNA [7]. Cells with severe damaged DNA will not commit to S phase. It has been reported that the presence of cells with unrepaired DNA in S phase of the cell cycle will initiate carcinogenesis [47,48].

Our findings showed that treatment with Gelam honey attenuated radiation-induced cell death by maintaining the expression of ATM gene when cells were treated before, during or after the irradiation process. The expression of ATM protein in HDFs decreased with Gelam honey treatment before, during and post-irradiation process. Similar up-regulation and down-regulation of *p53* gene was observed in irradiated HDFs and irradiated HDFs pre-treated with Gelam honey respectively. Our protein study however detected other group member of p53 which was p73 by the same antibody used in the research methodology. Similar finding has been reported by Turpeinen et al. which indicated that the same antibody can also detect other p53 group members such as p63 and p73 [49]. Studies done by Chen at al. reported that p73 was regulated by DNA damage and p53 [14]. It was also reported that p73 shared the same pathway with p53 and can trigger cell cycle arrest or induced apoptosis [15].

Gelam honey treatment was found to down-regulate p73 protein expression and allowed HDFs to proceed to S phase when the cells were treated before and during the irradiation process. Although both treatments resulted to progression of cell cycle to S phase, only pre-treated HDFs showed decreased induction of apoptosis while no changes was observed in HDFs treated during-irradiation when compared to irradiated HDFs. This observation may indicate inhibition of radiation-induced DNA damage by Gelam honey pre-treatment. These results are in line with our previous findings which showed decreased DNA damage in irradiated HDFs pre-treated with Gelam honey followed by increased in cell survival [38] indicating the progression of cell cycle and promotion of cell proliferation. Treatment with Gelam honey during and after the irradiation process however did not produce a significant protection against radiation-induced cell death. Although Gelam honey contains several antioxidant active compounds [26,35], natural existence of H_2_O_2_ radicals in honey may prevent the repairing process of damaged DNA. The accumulation of free radicals produced by ionizing radiation and H_2_O_2_ from Gelam honey may exceed the oxidative balance in the cells which eventually leads to increased oxidative stress and damaged the DNA molecules. Our previous study on antioxidant enzymes in HDFs showed that neither catalase nor glutathione peroxidase activities was increased in HDFs treated with Gelam honey during- or post-irradiation [39].

The expression of p16 gene and its translated protein was increased when HDFs were exposed to 1 Gy of gamma-rays indicating inactivation of cyclin D1-cdk4/6 complex. Similarly c*yclin D1* gene was up-regulated in irradiated HDFs and down-regulated with Gelam honey treatment. This up-regulation however was not followed by increased in cyclin D protein expression.

Since p16 shares the same mechanisms with p53 in inducing cell cycle arrest, the findings from this study may indicate that cell cycle arrest is also initiated by p73. This observation is in agreement with previous report by Leong et al. (2009) which showed that the expression of p16^ink4a^ depends on p53 expression [17]. HDFs treated with Gelam honey during irradiation however showed down-regulation of ATM, p53 and p16 genes, subsequently down-regulated cyclin D1 gene and allowed cell population to enter S phase. This scenario might explained the increased of cell survival rate in HDFs treated with Gelam honey observed in our previous report [38].

As for the expression of *GADD45* gene, we did not observe any significant changes with gamma-radiation and Gelam honey treatment. This finding may indicate that GADD45 is not involved in the cascade which induced cell cycle arrest and apoptosis of HDFs exposed to gamma-irradiation. The primer used in this study detected *GADD45γ* gene. Previous study reported that *GADD45γ* gene was activated by interleukin-6 and interleukin-2, whereas ionizing radiation activated GADD45α [50]. Similar results were observed for *PAK2* gene. According to Roig & Traugh (1999), PAK2 was expressed in fibroblast mice cells when exposed to gamma-rays [51] and its expression is highest 2 h after the radiation exposure, whereas in this study, the expression of *PAK2* gene was determined 24 h after radiation exposure. In summary, gamma-irradiation induced cell cycle arrest and apoptosis while treatment with Gelam honey promoted cell cycle progression and inhibited apoptosis. Treatment of Gelam honey prior to radiation exposure provide the best effect against ionizing radiation while treatment during and post-radiation exposure may not be beneficial.

## Conclusion

Gamma-irradiation up-regulated the expression of genes and proteins involved in the regulation of cell cycle subsequently initiated cell cycle arrest and induced apoptosis in HDFs. Gelam honey attenuated radiation-induced cell death by promoting cell cycle progression and inhibiting apoptosis indicating its molecular mechanism as a radioprotector against radiation damages.

## Competing interests

The authors declare that they have no competing interests.

## Authors’ contributions

SM was the Principal Investigator who designed the study and revised the manuscript. TAFTA carried out the lab work and drafted the manuscript. FJ, ZJ, KAR and NR were involved in data acquisition and revising the manuscript. All authors read and approved the final manuscript.

## Pre-publication history

The pre-publication history for this paper can be accessed here:

http://www.biomedcentral.com/1472-6882/14/108/prepub
